# Spinal cord infarction without radiologic evidence of trauma complicated by cerebellar infarction due to vertebral artery thrombosis: A case report

**DOI:** 10.1016/j.tcr.2026.101358

**Published:** 2026-04-27

**Authors:** Kazuki Hirose, Yuichi Saisaka, Ryo Ugawa, Yoshihiro Fujiwara, Maki Fukuda, Atsushi Morizane, Kouhei Miyashita, Toshiyuki Matsumoto

**Affiliations:** a Department of Orthopaedic Surgery, Kochi Health Sciences Center, Kochi, Japan; b Department of Emergency and Critical Care Medicine, Kochi Health Sciences Center, Kochi, Japan; c Department of Neurosurgery, Kochi Health Sciences Center, Kochi, Japan

**Keywords:** Spinal cord injury without radiographic evidence of trauma, Vertebral artery thrombosis, Cervical spinal cord injury, Cerebellar infarction, Multidisciplinary management

## Abstract

**Background:**

Spinal cord injury without radiographic evidence of trauma (SCIWORET) is a rare condition but remains relevant in elderly patients. Herein, we report the rare case of a 78-year-old woman who sustained a spinal cord injury after falling from a bicycle.

**Case report:**

Computed tomography (CT) at admission revealed an absence of bony abnormalities; however, motor and sensory deficits were observed below the C4 level, corresponding to Frankel grade B. Magnetic resonance imaging (MRI) demonstrated T2-weighted hyperintensity at levels C4–C7, indicative of spinal cord edema. Notably, axial MRI images revealed a loss of flow voids in the right vertebral artery, while contrast-enhanced CT identified a thrombotic occlusion accompanied by subsequent cerebellar infarction. Decompression surgery and coil embolization of the vertebral artery were accordingly performed. Despite early surgical intervention, no change in neurological status was observed, indicating complete spinal cord injury. The patient is consequently undergoing management in an intensive rehabilitation program focusing on optimizing respiratory function and preventing secondary complications.

**Conclusion:**

This case underscores the diagnostic value of flow void loss on axial MRI, which serves as a crucial indicator in the evaluation of patients with SCIWORET.

## Highlights

• Spinal cord injury without radiographic evidence of trauma (SCIWORET) is rare.
• We report a 78-year-old woman who sustained SCIWORET after a bicycle fall.
• Initial CT revealed no evidence of spinal cord injury.
• Subsequently, axial MRI showed a loss of flow voids in the right vertebral artery.
• Contrast-enhanced CT showed a thrombotic occlusion with cerebellar infarction.

## Introduction

Spinal cord injury without radiographic evidence of trauma (SCIWORET) is a rare but clinically significant condition characterized by neurological deficits without any identifiable vertebral fractures or dislocations on conventional radiographs or computed tomography (CT) scans [1]. This condition is most commonly observed in elderly individuals with preexisting cervical spinal canal stenosis or spondylotic changes, in whom even relatively minor trauma can result in significant spinal cord dysfunction [2]. The term ‘SCIWORET’ was initially introduced to differentiate such presentations from traditional cord injuries, which are typically associated with osseous disruption [3]. This condition is generally attributed to hyperextension mechanisms that generate transient, high-energy forces capable of compressing the spinal cord against a preexisting stenotic canal. Such mechanisms may result in spinal cord contusion, edema, or ischemia, even in the absence of any direct bony compression. Given the lack of overt skeletal injury, early diagnosis is often challenging and requires a high index of suspicion and prompt MRI to confirm cord damage [4]. MRI findings typically include T2-weighted hyperintensity, indicating cord edema or hemorrhage, which is essential to confirm the diagnosis and guide appropriate management.

In addition to mechanical injury, the potential of concomitant vascular complications must be considered in SCIWORET, particularly following high-energy trauma. Although rare, vertebral artery thrombosis (VAT) can occur in this context, and has been associated with posterior circulation infarcts, including cerebellar infarction. Such vascular complications are commonly underrecognized in acute trauma settings, where clinical attention is typically directed toward assessing spinal stability and preserving the neurological cord [5].

In this case report, we present a highly unusual case of SCIWORET complicated by vertebral artery thrombosis and cerebellar infarction, both identified through contrast-enhanced imaging. Contrast-enhanced CT is not routinely performed in patients with nontraumatic cervical spinal cord injury. However, in the present case, the absence of the expected flow void on axial MRI enabled the early detection of vertebral artery thrombosis, even prior to the performance of contrast-enhanced CT. This finding highlights the diagnostic value of meticulously evaluating axial MRI sequences for flow void defects, particularly when vertebral artery thrombosis is within the differential diagnosis. A flow void refers to the characteristic signal loss observed in flowing blood on T2-weighted images, the absence of which may indicate impaired flow or vascular occlusion. The coexistence of spinal cord injury and vascular complications represents a clinically significant scenario that necessitates comprehensive evaluation and coordinated multidisciplinary management.

## Case presentation

A 78-year-old woman with a medical history of hypertension was transported to the emergency department after falling from a bicycle. The mechanism of injury was presumed to involve hyperextension of the cervical spine. Prior to the incident, the patient was able to conduct activities of daily living (ADLs) completely independently and had no history of neurological deficits [1].

Upon admission, the patient was alert and oriented, with a Glasgow Coma Scale score of 15 (E4V5M6). However, neurological examination revealed quadriplegia consistent with a Frankel Grade B spinal cord injury. Manual muscle testing demonstrated preserved strength (MMT 3/3) only in the biceps brachii, whereas all other upper and lower extremity muscles were completely paralyzed (MMT 0/0). Sensory examination indicated a complete loss of sensation below the C4 level; however, perianal sensation was intact, and voluntary anal contraction was preserved. Abdominal-type breathing, suggestive of diaphragmatic paralysis, was noted, although hemodynamic stability was maintained despite marked bradycardia [2]. CT of the cervical spine revealed no evidence of any fracture or dislocation; however, ossification of the posterior longitudinal ligament was observed. The clinical presentation and neurological findings were consistent with SCIWORET. Magnetic resonance imaging (MRI) demonstrated a T2-weighted hyperintensity signal extending from the C4 to C7 levels, indicating spinal cord edema and contusion without any evidence of complete transection. No significant disc herniation was observed, further supporting the diagnosis of SCIWORET [4]. Notably, axial MRI images revealed a loss of the expected flow void in the left vertebral artery, indicating compromised blood flow.

**Fig. 1:**
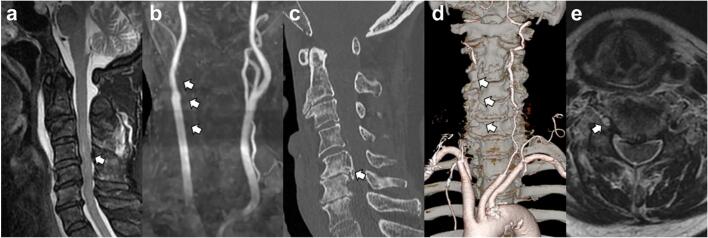
Imaging findings of the cervical spine and vertebral artery occlusion. (a) Sagittal T2-weighted fat-suppressed MRI demonstrating hyperintensity from C4 to C7, consistent with spinal cord edema and posterior soft tissue injury (arrow). (b) MRA revealing complete occlusion of the right vertebral artery (arrow). (c) Sagittal non-contrast CT depicting cervical canal stenosis due to the ossification of OPLL (arrow). (d) Contrast-enhanced CT confirming occlusion of the right vertebral artery (arrow). (e) Axial T2-weighted fat-suppressed MRI at the C5 level showing a normal flow void in the left vertebral artery and loss of the expected flow void in the right vertebral artery, suggestive of thrombosis (arrow).

**Fig. 2:**
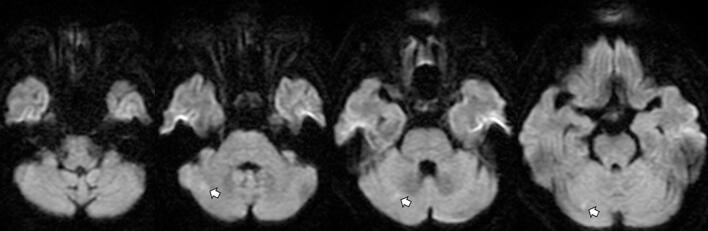
Diffusion-weighted magnetic resonance imaging showing acute cerebellar infarction. Diffusion-weighted MRI showing high signal intensity in the cerebellum, consistent with an acute infarction (arrow).

Given the high-energy mechanism of the injury, as well as the absence of fractures or dislocations, further vascular evaluation was deemed necessary. CT angiography (CTA) of the neck revealed complete occlusion of the right vertebral artery, which was confirmed by subsequent magnetic resonance angiography (MRA), which demonstrated an absence of flow in the right vertebral artery but preserved flow in the contralateral vessel. Brain MRA further revealed an acute infarction in the right cerebellum, consistent with vertebrobasilar ischemia. Notably, the patient did not exhibit any clinical signs of posterior circulation stroke, such as dysarthria, nystagmus, or ataxia, likely due to the dominant spinal cord deficits masking these manifestations [5] (Fig. 1, Fig. 2).

**Fig. 3:**
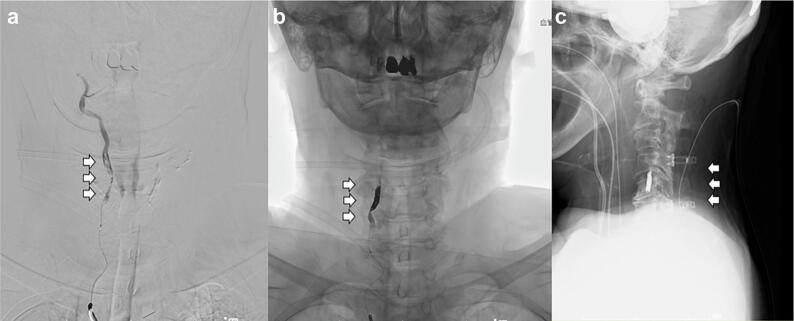
Postoperative radiographic images and coil embolization. (a) Intraoperative angiographic image taken during coil embolization showing irregular contrast opacification at the C4–C7 levels, suggestive of intimal injury to the vertebral artery (arrow). (b) Post-embolization angiogram confirming complete occlusion of the left vertebral artery (arrow). (c) Postoperative lateral radiograph demonstrating stable spinal alignment following C4–C7 laminoplasty (arrow).

Given the critical nature of the above vascular findings, emergency coil embolization of the right vertebral artery was performed to prevent any further embolic events and potential extension of cerebellar infarction. The procedure was conducted without complications, and complete occlusion of the affected vessel was achieved. This was followed by C4–C7 laminoplasty to decompress the spinal cord and stabilize the cervical spine, thereby addressing the suspected ischemic etiology of the cord injury (Fig. 3). Postoperatively, the patient was extubated and transferred to the intensive care unit to undergo neurological monitoring [6].

Despite early surgical intervention, the patient's neurological status remained unchanged postoperatively, which is consistent with a complete spinal cord injury. Currently, the patient continues to participate in an intensive rehabilitation program focusing on optimizing respiratory function and preventing secondary complications, such as deep vein thrombosis, pressure ulcers, and autonomic dysreflexia.

**Fig. 4:**
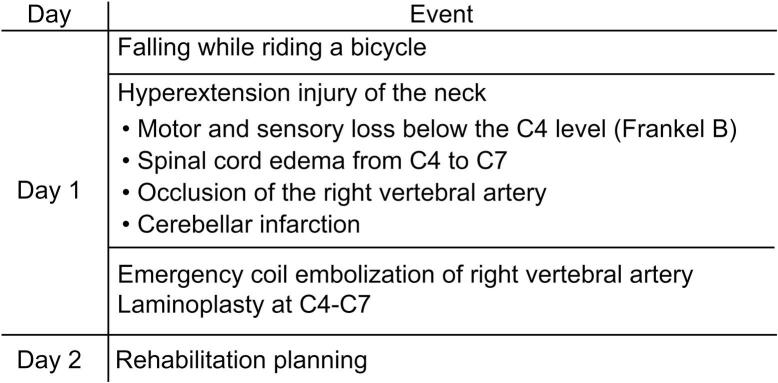
Timeline of clinical events in a case of SCIWORET with vascular findings. Chronological summary of the clinical findings and interventions, including the diagnosis of SCIWORET, development of vertebral artery thrombosis and cerebellar infarction, and emergency treatment.

No delayed neurological symptoms attributable to cerebellar infarction were observed throughout follow-up (Fig. 4).

## Discussion

Although SCIWORET is traditionally defined as a spinal cord injury without any radiographic evidence of trauma, this definition primarily refers to the absence of any significant fractures, dislocations, or spinal instability that may account for neurological deficits.

SCIWORET represents a rare but clinically significant subset of spinal cord injuries that typically occur in patients with preexisting spinal canal stenosis or degenerative spondylotic changes [7]. Unlike traditional spinal cord injuries that involve vertebral fractures or dislocations, SCIWORET is characterized by significant neurological impairment without any overt osseous disruption detectable on conventional imaging modalities, such as X-ray or CT scans [8]. This diagnostic ambiguity poses unique diagnostic and therapeutic challenges, as the absence of a visible bony injury can delay diagnosis and intervention [9].

The pathophysiology of SCIWORET is primarily attributed to the transient hyperextension or axial loading forces that compress the spinal cord against the stenotic spinal canal, resulting in contusions, edema, or ischemia. These mechanisms may lead to severe neurological impairment in elderly patients with longstanding cervical spondylosis, even following relatively minor trauma. MRI is essential to confirm the diagnosis by demonstrating T2-weighted hyperintensities indicative of cord edema or hemorrhage, as was observed in the present patient [10].

In our patient, the clinical course was further complicated by vertebral artery thrombosis—a rare but significant finding that significantly influenced prognosis and management [11]. Vertebral artery thrombosis is typically associated with high-energy trauma involving direct vascular injury, such as fractures or dislocations affecting the transverse foramen. However, in SCIWORET, thrombosis may occur in the absence of bony disruption. This is presumed to result from extreme rotational or hyperextension forces that stretch the vertebral artery or cause intimal damage. In the present patient, complete occlusion of the right vertebral artery—confirmed by CTA and MRA—likely contributed to the concurrent cerebellar infarction, as demonstrated by MRI [9].

The cerebellar infarction observed in this patient underscores the critical importance of early vascular imaging in patients with SCIWORET when posterior circulation compromise is suspected. Given the proximity of the vertebral arteries to the spinal column, even minor cervical trauma may result in significant vascular injuries, leading to posterior fossa infarction [10]. In this context, the lack of any overt cranial symptoms, such as dysarthria, nystagmus, or ataxia, may be attributed to dominant spinal cord deficits that mask these classic cerebellar signs.

Contrast-enhanced CT is not routinely performed in typical cases of SCIWORET. However, in the present patient, the absence of a normal flow void in the axial MRI sequences raised the suspicion of vertebral artery compromise. This finding prompted further vascular evaluation, including contrast-enhanced CT, brain MRI, and MRA, which confirmed occlusion of the right vertebral artery and guided the decision to proceed with coil embolization. This case underscores the critical diagnostic value of axial MRI in detecting vertebral artery compromise [12].

Furthermore, the absence of a normal flow void on T2-weighted MRI sequences could serve as an important imaging marker for detecting vertebral artery occlusion. Indeed, Ahn et al. recently reported that, among trauma patients, the loss of T2 flow voids in the vertebral arteries was strongly associated with vascular occlusion and subsequent brain infarction, even in the absence of apparent bony injury [13]. Their findings indicate that careful evaluation of axial T2-weighted images may allow early detection of traumatic vertebral artery compromise and guide timely vascular imaging.

The patient in the present case was managed through a multidisciplinary approach, including emergency vertebral artery embolization and cervical laminoplasty, which collectively addressed both the vascular and neurological components of her condition [9]. This case emphasizes the importance of early vascular imaging and coordinated care in the management of complex cervical cord injuries [11].

Overall, this case expands the current understanding of SCIWORET by illustrating an atypical presentation involving concurrent vertebral artery thrombosis and cerebellar infarction. Comprehensive imaging and multidisciplinary management are essential for delineating the full extent of injury. The inclusion of contrast-enhanced vascular imaging should be strongly considered in cases of high-energy cervical trauma to facilitate accurate diagnosis and timely intervention. In situations where contrast-enhanced studies are not initially obtained, meticulous assessment of axial MRI sequences for the absence of normal flow voids may provide critical diagnostic insight into underlying vascular compromise.

## Conclusions

This case illustrates the complex and multifaceted nature of SCIWORET, particularly when complicated by concurrent vascular pathology. The coexistence of SCIWORET, vertebral artery, and cerebellar infarction represents an unusual clinical scenario that challenges both conventional diagnostic and therapeutic approaches. The early recognition of SCIWORET requires a high index of suspicion, particularly among elderly patients with known spinal stenosis or degenerative changes who present with neurological deficits, even after minor trauma. In such cases, MRI remains the gold standard for identifying spinal cord edema or contusions, whereas vascular imaging is essential for detecting concomitant cerebrovascular complications. This case underscores the importance of comprehensive imaging—including contrast-enhanced modalities—to accurately delineate the full extent of neurological and vascular injuries. Furthermore, this case highlights the diagnostic value of axial MRI in detecting vertebral artery flow void defects. The early detection of such abnormalities may significantly alter the diagnostic pathway and could facilitate timely vascular management in complex presentations of SCIWORET [12].

In conclusion, this case report emphasizes the critical role of multidisciplinary care in the management of patients with complex trauma, highlighting the need for further investigation into the pathophysiological mechanisms underlying vascular injury in the context of SCIWORET. Such insights are essential for improving both the diagnostic accuracy and therapeutic outcomes in this challenging patient population.

## Ethics statements

This case report did not require approval from an institutional review board as it did not involve human research subjects. Written informed consent was obtained from the patient's family for the publication of this case report and accompanying images. All identifying information was anonymized to ensure the patient's confidentiality in accordance with ethical guidelines.

## CRediT authorship contribution statement

**Kazuki Hirose:** Conceptualization, Data curation, Investigation, Methodology, Project administration, Validation, Writing – original draft, Writing – review & editing. **Yuichi Saisaka:** Data curation, Investigation, Validation, Writing – review & editing. **Ryo Ugawa:** Formal analysis, Investigation, Methodology, Visualization, Writing – original draft, Writing – review & editing. **Yoshihiro Fujiwara:** Investigation, Supervision, Validation, Writing – review & editing. **Maki Fukuda:** Investigation, Writing – review & editing. **Atsushi Morizane:** Investigation, Writing – review & editing. **Kouhei Miyashita:** Investigation, Writing – review & editing. **Toshiyuki Matsumoto:** Resources, Supervision.

## Funding

This research did not receive any specific grant from funding agencies in the public, commercial, or not-for-profit sectors.

## Declaration of competing interest

The authors declare that they have no known competing financial interests or personal relationships that could have appeared to influence the work reported in this paper.

## Glossary

Spinal cord injury without radiographic evidence of trauma (SCIWORET): A spinal cord injury occurring in the absence of fractures, dislocations, or spinal instability on conventional radiographic imaging.
Flow void: A signal loss on MRI that normally represents rapidly flowing blood within a vessel, wherein loss of the normal flow void suggests vascular occlusion.
Vertebral artery thrombosis: A condition characterized by blood clot formation within the vertebral artery, causing partial or complete obstruction of blood flow and potentially resulting in ischemia of the posterior circulation, including the brainstem and cerebellum.
Cerebellar infarction: A type of ischemic stroke caused by reduced or blocked blood flow to the cerebellum, leading to tissue necrosis. Symptoms may include vertigo, ataxia, dysarthria, and impaired coordination.

## References

[1] Fehlings M.G., Vaccaro A., Wilson J.R. (2012). Early versus delayed decompression for traumatic cervical spinal cord injury: results of the surgical timing in acute spinal cord injury study (STASCIS). PLoS One.

[2] Vaccaro A.R., Hulbert R.J., Patel A.A. (2007). The subaxial cervical spine injury classification system: a novel approach to recognizing the importance of morphology, neurology, and integrity of the disco-ligamentous complex. Spine.

[3] Aarabi B., Albrecht J.S., Simard J.M. (2021). Trends in demographics and markers of injury severity in traumatic cervical spinal cord injury. J. Neurotrauma.

[4] Zmurko M.G., Tannoury T.Y., Tannoury C.A., Anderson D.G. (2003). Cervical sprains, disc herniations, minor fractures, and other cervical injuries in the athlete. Clin. Sports Med..

[5] Johnson V.E., Stewart W., Smith D.H. (2012). Widespread τ and amyloid-β pathology many years after a single traumatic brain injury in humans. Brain Pathol..

[6] Bok A.P., Peter J.C. (1996). Carotid and vertebral artery occlusion after blunt cervical injury: the role of MR angiography in early diagnosis. J. Trauma.

[7] Furlan J.C., Fehlings M.G. (2009). The impact of age on mortality, impairment, and disability among adults with acute traumatic spinal cord injury. J. Neurotrauma.

[8] Escario J.A., Sebastián C.S., Vizán A.A., Martínez-Quiñones J.V., Consolini F., Arregui Calvo R. (2017). Spinal cord injury and normal neuroimaging. Aetiology, diagnosis and medico-legal issues. Rev. Esp. Med. Legal.

[9] Takahashi M., Harada Y., Inoue H., Shimada K. (2002). Traumatic cervical cord injury at C3-C4 without radiographic abnormalities: correlation of magnetic resonance findings with clinical features and outcome. J. Orthop. Surg. (Hong Kong).

[10] Alvarez Reyes A., Hurlbert R.J., Dumont T.M., Ramey W.L. (2022). The number of organ system injuries is a predictor of intrahospital mortality in complete cervical spinal cord injury. World Neurosurg..

[11] Oyinbo C.A. (2011). Secondary injury mechanisms in traumatic spinal cord injury: a nugget of this multiply cascade. Acta Neurobiol. Exp. (Wars).

[12] Cox M., Kung D., Hurst R.W., Bagley L.J., Nabavizadeh S.A. (2019). Significance of the absent vertebral artery T2 flow void on cervical spine MRI in atraumatic patients without acute neurological symptoms. Neuroradiol. J..

[13] Ahn T.R., Kim S.W., Kang D.H. (2024). Absence of T2 flow voids in the vertebral arteries on cervical spine MRI as a marker of traumatic occlusion and brain infarction. Eur. J. Radiol..

